# Effect of Boiling Treatment on Linoleic Acid-Induced Oxidation of Myofibrillar Protein in Grass Carp

**DOI:** 10.3390/foods13244153

**Published:** 2024-12-22

**Authors:** Mengcong Liu, Fuhua Li, Yuan Tang, Jichun Zhao, Xiaojuan Lei, Jian Ming

**Affiliations:** 1 College of Food Science, Southwest University, Chongqing 400715, China; liumcong@163.com (M.L.); fuhualee92@163.com (F.L.); tangyuan925food@163.com (Y.T.); jichunzhao@swu.edu.cn (J.Z.); xjuanlei@swu.edu.cn (X.L.); 2 Research Center of Food Storage & Logistics, Southwest University, Chongqing 400715, China; 3 Chongqing Key Laboratory of Speciality Food Co-Built by Sichuan and Chongqing, Chongqing 400715, China

**Keywords:** boiling treatment, linoleic acid, myofibrillar protein, oxidation, water retention

## Abstract

The aim of this study was to investigate the promotion of linoleic acid (OLA)-induced myofibrillar protein (MP) oxidation by boiling treatment. The effect of the boiling treatment on grass carp MP oxidation induced by OLA was investigated. The total sulfhydryl content, fluorescence intensity, and amino acid content were reduced with the increasing OLA concentration after the boiling treatment, while the boiled oxidized MP’s carbonyl content (4.76 ± 0.14 nmol/mg) was 2.14 times higher than that of the native MP (2.22 ± 0.02 nmol/mg) at an OLA concentration of 10 mM. Additionally, the secondary structure of MP became more disordered, shifting from an α-helix to random coils and β-turns. When the concentration of OLA was higher than 5 mM, both the surface hydrophobicity and water holding capacity (WHC) decreased with the increasing OLA concentration. Furthermore, the boiling treatment led to a reduction in immobile water and an increase in free water content in the MP gel. These findings establish a theoretical basis for regulating MP oxidation to improve fish quality during boiling.

## 1. Introduction

Myofibrillar protein (MP) constitutes the predominant fraction of fish muscle protein, accounting for approximately 50–55% of the total [1]. It plays a crucial role in determining the structural integrity and functional properties of muscle proteins, including water retention [2] and gel characteristics [3], thereby influencing the texture and flavor of fish. The main constituents of MP, such as myosin heavy chain/light chain and actin, are highly abundant in fish muscle and particularly susceptible to oxidation [4,5,6,7]. Research indicates that oxidation of MP alters the physicochemical characteristics [8], thereby significantly impacting the quality and nutritional value of fish muscle [9,10].

Fish, as a vital source of high-quality protein, provide essential amino acids, micronutrients, and polyunsaturated fatty acids, contributing to the daily nutritional needs of humans [11]. However, during storage and processing, the presence of pro-oxidants, like unsaturated fatty acids and transition metal ions, renders MP highly vulnerable to oxidation [12,13]. This oxidative degradation leads to the loss of essential nutrients and causes undesirable changes in texture, WHC, color, and flavor while also promoting the formation of toxic substances. Given the pivotal role of MP in determining fish muscle quality, understanding the mechanisms and effects of MP oxidation is essential for mitigating quality deterioration and enhancing the storage, processing, and nutritional attributes of fish products.

Unsaturated fatty acids (UFAs) play an important role in the nutrition and flavor of foods [14,15]. UFAs are susceptible to oxidation, generating free radicals and reactive oxygen species (ROS) [16,17], which attack the amino acid residues on protein molecules, further inducing protein oxidation, resulting in structural and functional changes in proteins [18,19]. In grass carp, linoleic acid (OLA) serves as the primary source of polyunsaturated fatty acids [20]. As a widely used UFA, OLA has also been utilized to construct lipid-induced protein oxidation models. Jiang et al. [21] investigated the effect of OLA addition on the water holding capacity (WHC) of beef MP gel and indicated that a moderate concentration of OLA (≤6 mM) could increase the gel WHC, while a high concentration of OLA (>10 mM) led to protein aggregation, thereby reducing the gel WHC. Liao et al. [22] explored the effects on the structure and rheological properties of carp MP gel under the addition of OLA and showed that the OLA moderate oxidative modification (≤2.5 mM) enhanced the gel-forming ability of myofibrillar protein (MP). OLA with a concentration below 3 mM markedly increased the digestion rate and digestibility of MP, while at high concentrations (5–10 mM), OLA induced the adverse effect in protein digestion [23].

Current studies on MP oxidation induced by OLA are mostly conducted under ambient temperature conditions. However, fish are usually subjected to heat treatment before consumption, which inhibits the growth of microorganisms [24], enhances the organoleptic quality [25], as well as induces protein denaturation, facilitating digestion and absorption [26]. Despite these benefits, heat treatment also leads to protein oxidation, which can negatively impact the fish meat quality and nutritional value [27]. Yu et al. [28] used a simulated digestion model to study the impact of oxidation on protein digestion in cooked abalone muscle. The results indicated that the boiling treatment caused protein oxidation in abalone muscle, significantly inhibiting the degree of hydrolysis and digestibility of the proteins. Zhang et al. [29] extracted oyster salt-soluble proteins (OSSP) from Pacific oysters and examined the effects of heat treatment (35–100 °C) on the physicochemical properties and in vitro digestion of OSSP. The findings revealed that heating significantly reduced protein solubility and increased surface hydrophobicity and carbonyl content. In vitro digestion results demonstrated that protein oxidation and aggregation inhibited the digestion of OSSP [29]. Additionally, Xia et al. [30] investigated the effects of different roasting temperatures (150 °C, 190 °C, 230 °C, 270 °C, 310 °C) on protein oxidation and amino acid modifications in beef patties. The results showed that, as the roasting temperature increased, the level of protein oxidation intensified, amino acid side chains were modified, high temperatures significantly increased cooking loss, high temperatures elevated pH values, and high temperatures resulted in different color values. During heat treatment, the interaction between proteins and lipids can significantly influence protein oxidation. However, the impact of heat treatment on protein oxidation, specifically focusing on OLA-induced MP oxidation, remains insufficiently studied.

Therefore, grass carp MP was studied in this paper, and the heat treatment was applied following OLA-induced MP oxidation to investigate the impact and possible mechanisms of the heat treatment on the oxidation of grass carp MP. The changes in MP oxidation, thermal aggregation, and gel properties were analyzed by measuring the carbonyl and sulfhydryl contents, surface hydrophobicity, solubility, and WHC. The results may contribute to a deeper understanding of the oxidation mechanisms of MP under the dual influence of heat treatment and OLA oxidation, providing scientific guidance for protein protection and quality control during the heat processing of fish.

## 2. Materials and Methods

### 2.1. Samples and Materials

The grass carp were purchased from Yonghui Supermarket in Chongqing, and muscle tissues were collected and portioned into packages of 100 g each at temperatures between 0 and 4 °C. The linoleic acid was chromatographically pure (Macklin, Shanghai, China). 2,4-dinitrophenylhydrazine (DNPH), trichloroacetic acid (TCA), guanidine hydrochloride, and 5,5′-dithio-bis-(2-nitrobenzoic acid) (DTNB) were purchased from the Aladdin Chemistry Co., Ltd. (Shanghai, China). Tris-(hydroxymethyl)-aminomethane (Tris), tetramethylenediamine (TEMED), ammonium persulfate (APS), and glycine were obtained from the Sigma Chemical Co. (St. Louis, MO, USA). Coomassie brilliant blue R-250 was purchased from the Labgic Technology Co., Ltd. (Beijing, China). Bromophenol blue (BPB) and sodium dodecyl sulfate (SDS) were purchased from Kelong Chemical Reagent Co., Ltd. (Chengdu, China).

### 2.2. Experimental Methods

#### 2.2.1. MP Extraction

The extraction of MP was conducted by the method of a previous study, with slight modifications [31]. The grass carp meat (10.00 g) was added to 40 mL of pre-cooled 0.01 M phosphate buffer (containing 0.1 M NaCl, 0.002 M MgCl_2_, and 0.001 M EGTA, pH = 7.0) and homogenized at 10,000 rpm for 2 min. The sample was then centrifuged at 4000× *g* for 10 min at 4 °C. This process was repeated twice. The resulting precipitate was then resuspended in 40 mL of pre-cooled phosphate buffer (pH = 6.25) containing 0.1 M NaCl, homogenized at 10,000 rpm for 1 min, and centrifuged at 4000× *g* for 10 min. This was repeated twice. The final centrifugation was performed by adding 40 mL of pre-cooled phosphate buffer containing 0.1 M NaCl (pH = 6.0) to the last precipitate, homogenizing at 10,000 rpm for 1 min, filtering through four layers of gauze, discarding the precipitate and retaining the supernatant, and centrifuging at 8000 rpm for 10 min. The resulting precipitate was MP. The extracted MP was dispersed into 0.02 M phosphate buffer solution (pH = 6.0, containing 0.6 M NaCl). It was placed in storage at 4 °C and used within 48 h.

#### 2.2.2. MP Oxidation

The MP oxidation method was referenced and modified [21]. Different concentrations of OLA, 0, 2.5, 5, 7.5, and 10 mM, were added to the MP resuspension solution (10 mg/mL). The samples were incubated at 25 °C in a dark environment for 12 h. After the specified time, the protein was centrifuged at 4 °C for 15 min at 8000 rpm. Subsequently, the MP concentration was adjusted to the desired level and incubated in a boiling water bath for 5 min. The un-boiled MP was recorded as the control, and the 100 °C boiling water bath MP was recorded as the boiling group.

#### 2.2.3. Determination of Protein Oxidation

##### Carbonyl Content Measurement

The reaction of the proteins with DNPH was utilized to determine the carbonyl content of the proteins [17]. The MP solution (0.8 mL, 5 mg/mL) was mixed with 2 mol/L hydrochloric acid (1.6 mL, containing 1% DNPH). The reaction was carried out in the dark at room temperature for 1 h. Then, 2 mL of 40% TCA (*w*/*v*, 40 mg/100 mL) was added to the mixture to precipitate the proteins, which were incubated for 20 min and then centrifuged at 4 °C for 15 min at 8000 rpm. The precipitates were washed with 2 mL of ethanol:ethyl acetate (1:1, *v*/*v*) until the supernatant was colorless and clear. Finally, the precipitate was dissolved in 2 mL of phosphate buffer (pH = 6.5, containing 6 M guanidine hydrochloride) and incubated at 37 °C until the precipitate was completely dissolved. The absorbance of the solution at 370 nm was determined. The carbonyl content was calculated using the carbonyl molecular absorbance coefficient of 22,000 M^−1^cm^−1^, and the result was expressed as nmol of DNPH immobilized per mg of protein.

##### Total Sulfhydryl Content Measurement 

The method used to determine the sulfhydryl content was based on a previous study, with minor modifications [17]. A total of 1 mL of a 5 mg/mL MP solution was mixed with 9 mL of 0.05 M phosphate buffer (pH = 8.0, containing 8 M urea, 0.6 M NaCl, and 0.01 M EGTA). A total of 3 mL of the diluted solution was mixed with 0.4 mL of 0.001 M DTNB. The reaction was carried out at room temperature and protected from light for 1 h. The absorbance was measured at 412 nm.

(1) $$\mathrm{Total}\;\mathrm{sulfhydryl}\;(µ\mathrm{mol}/g\;\mathrm{protein})=\frac{A_{412}\times 10^{5}}{C\times 136}$$

where “*A*_412_” is the absorbance at 412 nm and “*C*” is the protein concentration (mg/mL).

##### Fluorescence Spectroscopy

A previously established method was referenced with slight modifications [31]. The MP concentration was adjusted to 1 mg/mL, and the fluorescence spectrophotometer was set to scan within the range of 300–400 nm, using an excitation wavelength of 295 nm and excitation/emission slit widths of 5 nm.

#### 2.2.4. Determination of Protein Thermal Aggregation Indices

##### Surface Hydrophobicity Determination

Surface hydrophobicity was determined according to a previous method [32], with some modifications. A total of 1 mL of a 5 mg/mL MP solution was mixed with 200 µL of a 1 mg/mL BPB solution and 200 µL of a 1 mg/mL BPB, and 1 mL 0.04 M phosphate buffer (pH = 6.8) was added to the control. Then, they were mixed well and reacted at room temperature for 10 min. After centrifuging at 8000× *g* for 15 min at 4 °C, 0.1 mL of the supernatant was diluted with 0.9 mL phosphate buffer, and the absorbance was measured at 595 nm. Surface hydrophobicity was expressed as the amount of BPB binding.

(2) $$\mathrm{BPB}\;\mathrm{bound}\;(µg)=\frac{200\times (A_{control-}A_{sample})}{A_{control}}$$

##### Particle Size and Zeta Potential

The MP particle size (d_50_) was determined using a Malvern Laser Particle Sizer 2000 [33]. Adjusting the MP concentration to 10 mg/mL, the relative refractive index and absorption were set as 1.414 and 0.001, respectively.

The concentration of the MP solution was adjusted to 1 mg/mL, and 1 mL of the MP solution was added to the zeta potential dish at room temperature. The zeta potential was tested with a 90° scattering angle, and the equilibration time was 60 s [31].

##### Turbidity and Solubility

Turbidity was measured at an absorbance of 370 nm using a 1 mg/mL MP solution [31].

Solubility was measured as described by a previous study, with slight modifications [31]. The concentration of the MP solution was adjusted to 1 mg/mL, and the protein concentration was measured at 562 nm. The MP solution was centrifuged at 8000× *g* rpm for 15 min at 4 °C, and the supernatant was collected for further analysis.

(3) $$\mathrm{MP}\;\mathrm{solubility}/\%=\frac{protein\;concentration\;after\;centrifugation/(\mathrm{mg}/\mathrm{mL})}{protein\;concentration\;before\;centrifugation/(\mathrm{mg}/\mathrm{mL})}100\%$$

#### 2.2.5. Determination of Protein Structure

##### Ultraviolet–Visible Absorption Spectroscopy

The concentration of MP was adjusted to 0.5 mg/mL, and the UV–visible absorption spectra were measured in the range of 230–340 nm.

##### Fourier Transform Infrared (FTIR) Spectroscopy

The oxidized MP samples were taken and lyophilized. The lyophilized MP powder was then accurately weighed, mixed with a potassium bromide standard, and pressed into tablets. These tablets were scanned over the full spectral range of 400–4000 cm^−1^, with 64 scans accumulated for each measurement. Protein secondary structure analyses were performed using PeakFit v4.12 software. The spectral regions near 1645–1660 cm^−1^, 1670–1680 cm^−1^, and 1660–1670 cm^−1^ correspond to α-helix, β-sheet, and random coil, respectively. In contrast, β-turns are characterized by peaks located around 1640–1645 cm^−1^ and 1680–1690 cm^−1^.

##### Scanning Electron Microscope (SEM)

The MP freeze-dried powder sample was glued to the sample stage, and platinum film was plated with an iron sputterer, with the film thickness of about 100 A. The test conditions were as follows: accelerating voltage was 10 kV, and representative surface microstructures were observed and photographed under 5000x magnification.

##### SDS-PAGE

The cross-linking extent of the proteins was determined by SDS-PAGE [22]. The concentration of MP was adjusted to 1 mg/mL and mixed with sample buffer in a 3:1 ratio. The mixture was boiled in a water bath for 5 min, cooled to room temperature, and centrifuged at 8000× *g* for 5 min. From the supernatant, 200 µL was taken for analysis. A 12% separating gel and a 5% stacking gel were used. Electrophoresis was carried out at 80 V. After electrophoresis, the gel was stained overnight and then decolorized with decolorizing solution until the liquid was colorless. The protein molecular weight was determined using a protein marker.

#### 2.2.6. Amino Acid Content Determination

The amino acid content was determined according to a previous study, with modifications [34]. An appropriate amount of freeze-dried powder was mixed with 10 mL of 6 M hydrochloric acid (containing 1% phenol), exposed to a nitrogen gas environment for 1 min, and finally hydrolyzed at 110 °C for 22 h. The hydrolyzed sample was diluted to 50 mL. A total of 1 mL of the above solution was blown dry with nitrogen; then, 1 mL of 0.01 M HCl was added to dissolve it, and it was measured through a 0.22 μm membrane. Finally, the filtered solution was tested by an automatic amino acid analyzer.

#### 2.2.7. Determination of MP Gel Water Retention

##### Preparation of MP Gel

MP gel was prepared based on a previously established method, with some modifications [35]. The extracted MP was adjusted to 40 mg/mL with a 0.02 M phosphate buffer solution (pH = 6.0, containing 0.6 M NaCl). After oxidation, MP was bathed at 80 °C for 1 h and cooled at 25 °C; then, the protein gel sample was obtained.

##### Water-Holding Capacity (WHC)

The WHC was calculated according to Equation (4) [36]. Approximately 3 g of MP gel was centrifuged at 8000× *g*, 4 °C for 15 min, and then the resulting weight was recorded.

(4) $$\mathrm{WHC}\;(\%)=\frac{W_{2}}{W_{1}}\times \;100\%$$

where *W*_1_ represents the weight before centrifugation and *W*_2_ represents the weight after centrifugation.

##### Low-Field Nuclear Magnetic Resonance (LF-NMR) Proton Relaxation

The T_2_ value (transverse relaxation time) of gel water was determined by an LF-NMR imaging analyzer [37]. The magnetic field strength was 0.5T at 32 °C, and the corresponding proton resonance frequency was 22.4 MHz. The other NMR parameters were as follows: SW = 250 kHz, SF = 23 MHz, RFD = 0.080 ms, RG1 = 10.0 db, P1 = 11.52 µs, DRG1 = 4, TD = 1,125,038, PRG = 2, TW = 3000.000 ms, NS = 8, PT = 23.52 µs, TE = 0.250 ms, and NECH = 18,000.

### 2.3. Statistical Analysis

Statistical analysis was performed using SPSS (Version 27.0) software, and one-way ANOVA was used for significant difference analysis (*p* < 0.05 indicates a significant difference). Protein secondary structure analysis was performed by PeakFit v4.12 software and plotted using Origin 2024 software (OriginLab Corporation, Northampton, MA, USA).

## 3. Result and Discussion

### 3.1. Protein Oxidation Analysis

#### 3.1.1. Carbonyl Content

**Figure 1:**
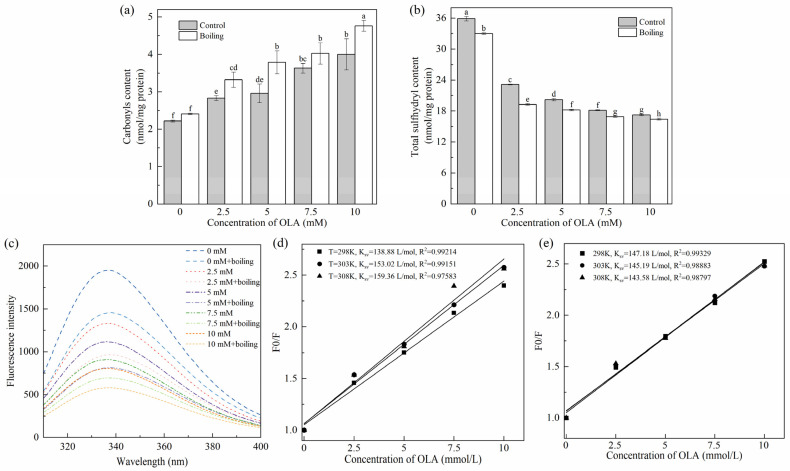
Effect of boiling treatment and OLA concentration on MP carbonyl content (**a**), total sulfhydryl content (**b**), and fluorescence intensity (**c**). Stern–Volmer curves of MP fluorescence quenching by OLA at different reaction temperatures: (**d**) un-boiled and (**e**) boiled for 5 min. Different capital letters (a–h) indicate a significant difference (*p* < 0.05) between the control and boiling samples at different OLA concentrations.

The protein carbonyl content was positively correlative to the degree of protein oxidation [38]. As shown in Figure 1a, before the boiling treatment, as the OLA concentration increased to 10 mM, the carbonyl content of MP increased significantly (*p* < 0.05) in a dose-dependent manner, and the maximum content was 1.80 times higher than that of the unoxidized MP (4.00 nmol/mg vs. 2.22 nmol/mg), indicating that the increase in OLA concentration exacerbated the MP oxidation. Similar results were found previously [23]. All the boiling-managed MP showed higher levels of carbonyl than those of the un-boiled at the same OLA concentration. The increasing degree of oxidation was associated with accelerated oxidation of OLA induced by heating; free radicals or aldehydes produced by oxidation of OLA would attack the amino acid side chains of proteins, causing excessive protein oxidation [8]. Hence, the boiling treatment promoted the oxidation of MP by OLA.

#### 3.1.2. Total Sulfhydryl Content

The reduction in total sulfhydryl content is a key indicator of protein oxidation in meat products. Research indicates that sulfhydryl groups in amino acids, such as methionine and cysteine, are highly susceptible to oxidation, leading to the formation of disulfide bonds. These sulfhydryl groups react readily with DTNB, producing yellow 4-nitrothiophenol compounds. As a result, the DTNB method, also known as Ellman’s method, is commonly employed to monitor changes in total sulfhydryl content in proteins by analyzing the color intensity of the resulting solution [39]. As can be seen in Figure 1b, the total sulfhydryl content of MP was significantly decreased (*p* < 0.05) after oxidation compared with unoxidized MP (0 mM). This value closely matched the results reported by Cheng et al. [17]. Furthermore, a higher degree of oxidation resulted in a further decrease in total sulfhydryl content. The reactive oxygen radicals and hydroperoxides generated by OLA peroxidation attacked the cysteine residues of sulfur-containing amino acids in MP, leading to the change in the primary structure of MP. This finding aligned with the conclusion reported by Zhang et al. [35] based on the Fenton oxidation model, which demonstrated the impact of H_2_O_2_ concentration on the sulfhydryl content of MP. The total sulfhydryl content of MP after the boiling treatment at the same concentration of OLA was significantly lower (*p* < 0.05) than that before boiling; the generation of intra- or inter-protein disulfide bonds and mixed disulfide bonds was used to explain it.

#### 3.1.3. Amino Acid Analysis

The contents of 17 amino acids are shown in Table 1. They decreased gradually with increasing OLA concentration before boiling. The MP amino acid content after oxidation treatment was significantly lower (*p* < 0.05) than that of the unoxidized samples, which was in alignment with the results of Zhang et al. [37]. The sulfur-containing amino acids cysteine and methionine had been used as degradation markers of proteins exposed to H_2_O_2_ oxidation in previous studies [40], indicating that they are sensitive to ROS produced by oxidation; the same results were found in our experiment. The continuous decrease in histidine content could explain the increasing carbonyl content (Figure 1a). In addition, lysine was easily oxidized by OLA products to form adducts, leading to a decrease in lysine content [41]. Negatively charged amino acids, such as aspartic acid and glutamic acid, also showed significant decreases in their contents (*p* < 0.05), which would have an effect on the zeta potential of MP. After boiling, the change trend of amino acid contents in MP with OLA concentration was the same as that before boiling, but at the same OLA concentration, the contents of 17 amino acids were lower than those before boiling. The results indicate that the high-temperature boiling treatment not only accelerated OLA oxidation but also altered the exposure level of amino acid residues, subsequently promoting OLA-induced oxidative degradation of proteins.

#### 3.1.4. Tryptophan Endogenous Fluorescence Intensity

The oxidation of tryptophan results in the formation of N-formylkynurenine and kynurenine [42], leading to a decrease in fluorescence intensity and a shift in the maximum wavelength. Additionally, tryptophan residues with high fluorescence intensity are typically located in the hydrophobic environment of natural proteins. During the denaturation of proteins, tryptophan residues become gradually accessible to the surface region, leading to a reduction in fluorescence [32]. Consequently, the reduction in the intensity of tryptophan endogenous fluorescence is the result of a combination of changes in protein structure and oxidation levels, which can be used to assess the degree of oxidation of proteins [27]. Figure 1c demonstrates the effect of the boiling treatment and OLA concentration on MP fluorescence intensity. Before the boiling treatment, the fluorescence intensity of MP decreased gradually (from 1952 to 806) with increasing OLA concentration. After the boiling treatment, the trend of MP fluorescence intensity with increasing linoleic acid concentration was consistent with that before the boiling treatment. At the same OLA concentration, the fluorescence intensities of MP after the boiling treatment were all lower than those before the boiling treatment. This indicates that the boiling treatment further modified the extent of OLA-induced oxidation of MP, resulting in MP denaturation and the exposure of tryptophan residues buried within the molecule to the external polar environment [27]. The exposure of hydrophobic residues enhanced intermolecular hydrophobic interactions and promoted the formation of aggregates, which resulted in a decrease in the intensity of the protein’s endogenous fluorescence [43].

The fluorescence quenching mechanism between MP and OLA was further analyzed using the Stern–Volmer Equation (5). Fluorescence quenching usually occurs through either dynamic quenching or static quenching mechanisms, which can be distinguished by their dependence on temperature [44].

(5) $$\frac{F_{0}}{F}=\;K_{\mathrm{SV}}\;[Q]$$

where *F*_0_ represents the fluorescence intensity of natural MP (OLA concentration of 0 mM) and *F* represents the fluorescence intensity of MP at different OLA concentrations. K_SV_ is the dynamic quenching constant, and [Q] is the OLA concentration.

As shown in Figure 1d,e, the fitted correlation coefficients (*R*^2^) of the Stern–Volmer equation before and after boiling were greater than 0.97 at all three temperatures, indicating that the equation fitted the data well. K_SV_ gradually increased with the increase in reaction temperature, indicating the existence of dynamic quenching of the fluorescence quenching mechanism of MP by OLA (Figure 1d). K_SV_ gradually decreased with the increase in reaction temperature, indicating that the fluorescence quenching mechanism of OLA on MP after the boiling treatment might be a static quenching; the complexes between the quenching agent and the fluorophore molecules were formed (Figure 1e). However, Kq (the rate constant of bimolecular quenching) was still smaller than the maximum dynamic quenching rate (2.0 × 10^10^ L/mol s), suggesting the co-existence of static and dynamic quenching [45]. It was evident that the high-temperature boiling treatment could enhance the collision and binding between MP and OLA/OLA’s oxidation products, thereby promoting the oxidation of MP induced by OLA.

### 3.2. Protein Thermal Aggregation Analysis

#### 3.2.1. Surface Hydrophobicity

**Figure 2:**
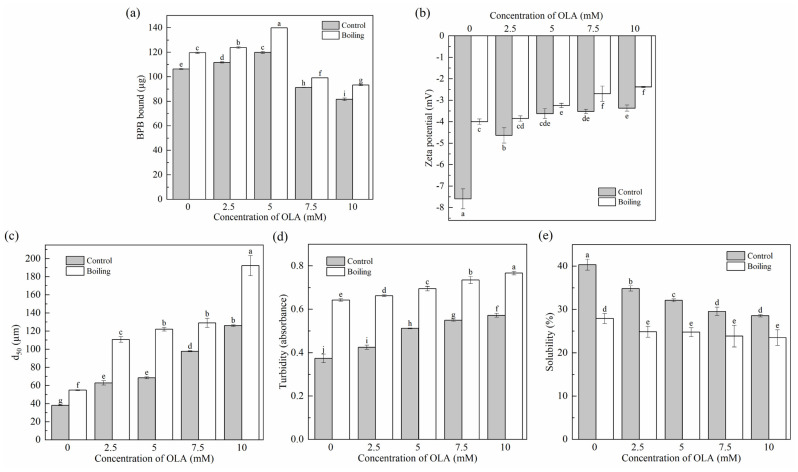
Effect of boiling treatment and OLA concentration on the surface hydrophobicity (**a**), zeta potential (**b**), particle size (**c**), turbidity (**d**), and solubility (**e**) of MP. Different capital letters (a–i) indicate a significant (*p* < 0.05) difference between the control and boiling samples at different OLA concentrations.

Changes in protein surface hydrophobicity were used to evaluate protein conformational changes, indicating the exposure of hydrophobic structures in proteins [46]. As shown in Figure 2a, the surface hydrophobicity of unoxidized MP was 106.28 ± 0.54 µg; the same value was reported by Liao et al. [22]. Before boiling, when the OLA concentration was lower than 5 mM, the surface hydrophobicity of MP increased significantly (*p* < 0.05) with the increase in OLA but decreased significantly (*p* < 0.05) with a further increase in OLA (>5 mM). Similar results were observed in previous studies [21,47]. The binding of OLA oxidation products to the exposed hydrophobic structures of MP might cause a decrease in the amount of BPB binding with MP hydrophobic structures [48]. The decrease in protein surface hydrophobicity was also associated with protein aggregation [49], the covalent modification of exposed hydrophobic residues (tryptophan residues) [50], and the generation of new hydrophilic components (protein carbonyls). All the surface hydrophobicity of boiled MP showed higher levels than that of the un-boiled MP, which was consistent with a previous study [51]. Hence, the boiling treatment increased the exposure of hydrophobic groups in MP caused by a certain concentration of OLA to improve the surface hydrophobicity of protein.

#### 3.2.2. Zeta Potential

Zeta potential reflected the electrostatic interactions between protein particles in the MP solution and was closely related to the stability of MP in solution. The smaller the absolute value of the zeta potential, the more aggregated the system was [52]. As shown in Figure 2b, the zeta potential of MP was always negative before and after boiling, which was related to the negatively charged glutamic acid and aspartic acid in the side chains of MP [53]. With the increase in OLA concentration, the decreased zeta potential was related to the oxidation of the negatively charged amino acid side chains (Section 3.1.3). In addition, the zeta potential after boiling was always lower than that without boiling. High oxidation intensity weakened the electrostatic repulsive force between the protein molecules and reduced the stability of MP in solution, resulting in the protein aggregation [54].

#### 3.2.3. Particle Size, Turbidity, and Solubility

Changes in the degree of protein cross-linking and aggregation were assessed by determining the average particle size d_50_, turbidity, and solubility of the MP samples.

As shown in Figure 2c, the d_50_ of MP significantly increased (*p* < 0.05) with increasing OLA concentration before and after boiling. This was due to oxidation-caused protein unfolding, exposing charged amino acid residues. These residues then reacted with OLA oxidation products to generate electrically neutral substances, weakening the electrostatic interactions between protein particles and increasing cross-linking and aggregation of protein molecules. As a result, the average particle size of the proteins increased. This was in agreement with the results of changes in tryptophan endogenous fluorescence intensity (Section 3.1.4) and zeta potential (Section 3.2.2).

The changes in MP turbidity and solubility are shown in Figure 2d,e. Before boiling, as the OLA concentration increased to 10 mM, the turbidity of MP gradually increased, and the solubility gradually decreased, showing an opposite trend between protein turbidity and solubility [55]. Boiled MP showed higher turbidity and lower solubility than un-boiled MP. This occurred because boiling induced protein denaturation, unfolding, and aggregation. The protein aggregate formation led to an enlarged diameter of the suspended particles in the solution, enhancing light scattering, increasing turbidity, and reducing solubility [56].

### 3.3. Protein Structure Analysis

#### 3.3.1. UV Absorption of MP

**Figure 3:**
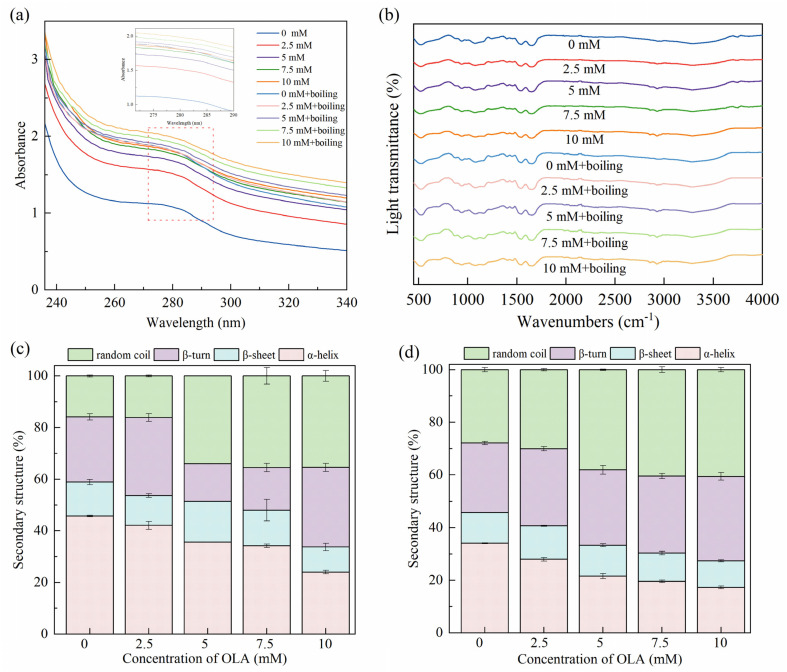
Effect of boiling treatment and OLA concentration on MP UV–visible absorption spectra (**a**), FTIR spectra (**b**), and secondary structure: (**c**) un-boiled and (**d**) boiled for 5 min.

Since the side chain groups of tryptophan and tyrosine residues can absorb UV light, UV absorption spectroscopy was used to study the changes in the tertiary structure of the proteins [57]. As shown in Figure 3a, the characteristic absorption peaks appearing around 280 nm wavelength of MP were slightly red-shifted and accompanied by an enhancement in UV absorption intensity after boiling and different OLA concentrations. This indicates that boiling and treatment with varying OLA concentrations exposed aromatic amino acid tryptophan and tyrosine residues to solvents, shifting them towards more hydrophilic environments. Consequently, the proteins underwent unfolding and denaturation [58]. Consistent results were also observed in the fluorescence spectra (Section 3.1.4) and surface hydrophobicity measurements (Section 3.2.1).

#### 3.3.2. Secondary Structure

FTIR was used to analyze the MP conformational changes induced by the boiling treatment and different OLA concentrations. As shown in Figure 3b, significant shifts and changes were not caused in the MP IR spectra with the boiling treatment and the different OLA concentrations, and the trends remained basically the same. The secondary structures presented in the propenamide I band (1600–1700 cm^−1^) were analyzed, and the results are shown in Figure 3c,d. Before boiling, the α-helix content in MP gradually decreased with the increasing OLA concentration. The stability of the α-helix was related to intermolecular hydrogen bonding [32]. Protein oxidation was caused by protein unfolding and destruction of intermolecular hydrogen bonding, resulting in a decreased α-helix content and transformation into an irregularly curled structure. Boiling further decreased the α-helix content while increasing the β-turn and random coil contents. Previous studies showed that heating reduced the α-helix content of proteins, leading to a change in protein structure from ordered to disordered [59,60].

#### 3.3.3. Microstructure

**Figure 4:**
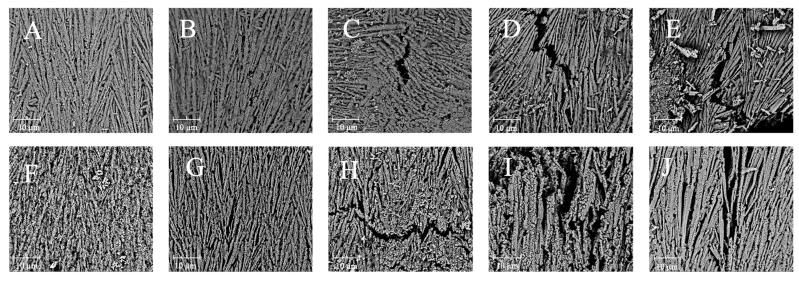
Effect of boiling treatment and OLA concentration on the microstructure of MP at 5000× magnification ((**A**,**F**): 0 mM, (**B**,**G**): 2.5 mM, (**C**,**H**): 5 mM, (**D**,**I**): 7.5 mM, (**E**,**J**): 10 mM. (**A**–**E**) was un-boiled; (**F**–**J**) was boiled for 5 min).

The microstructural changes in the boiling-treated and OLA-induced oxidized MP were determined by SEM. The microstructure of MP appeared fibrillar at 5000x magnification, which was consistent with the previous findings [61]. Similarly, MP’s microstructure was filamentous under an atomic force microscope [62,63]. Figure 4A–E illustrate the effects of varying OLA concentrations on MP’s microstructure before boiling. As the OLA concentration increased to 5 mM, the microstructure of MP became looser with larger pore sizes. At a higher OLA concentration (>5 mM), more gaps and a more fragmented structure were observed, indicating severe protein microstructural destruction. The effect of hydroxyl radical oxidation induction on squid MP gel microstructure was investigated by Nyaisaba et al. [46]; they found that the densification of the MP gel decreased with increasing hydrogen peroxide concentration, resulting in fragmented proteins and a less compact network. The microstructures of MP oxidized with various OLA concentrations after boiling are shown in Figure 4F–J. Compared to the un-boiled samples, the boiled samples exhibited larger protein pores and agglomeration, forming larger molecules, which was in line with earlier findings [29]. These results indicate that the boiling treatment exacerbated MP aggregation and OLA-induced protein microstructure changes.

#### 3.3.4. SDS-PAGE Analysis

**Figure 5:**
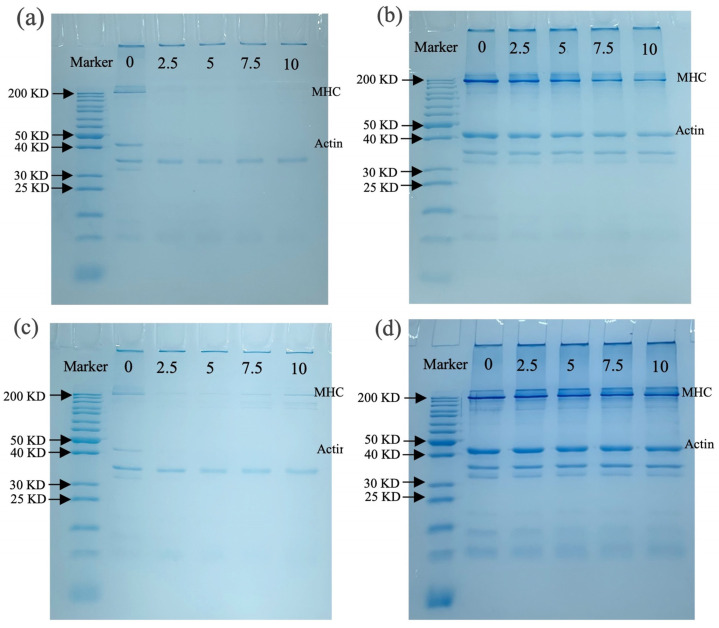
Effect of boiling treatment and OLA concentration on SDS-PAGE of MP: (**a**) un-boiled and in the absence of β-ME; (**b**) un-boiled and in the presence of β-ME; (**c**) boiled and in the absence of β-ME; (**d**) boiled and in the presence of β-ME. The lanes from left to right are standard and oxidized protein with 0, 2.5, 5, 7.5, and 10 mM OLA.

SDS-PAGE was used to detect aggregation, cross-linking, and molecular weight changes in MP after oxidation. Figure 5a shows the molecular weight changes in MP before boiling without β-ME, where the myosin heavy chain (MHC) and actin bands of MP almost disappeared with increasing OLA concentration. Similar results were observed in malondialdehyde-induced oxidation of pork MP, suggesting excessive cross-linking of MP occurred [60]. The presence of distinct bands at the top of the stacking gel also suggested that OLA caused cross-linking of MP, forming large protein aggregates that could not enter the stacking gel [21]. When containing β-ME (Figure 5b), most disappearing protein bands gradually recovered, especially MHC and actin, suggesting that MP polymers promoted protein cross-linking through disulfide bonds [35]. However, the loss of MHC and actin bands was not fully recovered under a high OLA concentration (>5 mM) compared to MP samples oxidized with a low concentration (≤ 5 mM), and the results suggest that non-disulfide bonds played an important role in MP band deletion under high-intensity oxidation [22].

Figure 5c shows protein molecular weight changes with the boiling treatment without β-ME; the MHC and actin bands of MP are lighter than those in Figure 5a. Under non-reductive conditions, more pronounced protein aggregation in the stacking gel was observed than in the un-boiled sample (Figure 5a), suggesting the boiling treatment enhanced MP cross-linking. A similar result was found by Chen et al. [64]. With β-ME (Figure 5d), a large protein band recovery was seen, and the changes in MHC and actin bands are consistent with Figure 5b. The richer protein bands observed in Figure 5d than in Figure 5b might be associated with the boiling treatment accelerating MP oxidation by OLA, resulting in protein degradation or breakage [60].

### 3.4. Analysis of Water Retention of MP Gel

**Figure 6:**
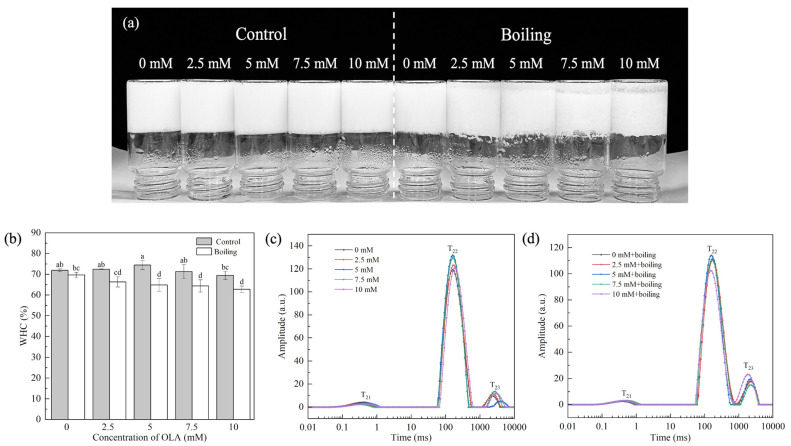
Effect of boiling treatment and OLA concentration on the macroscopic of MP gel (**a**). Effect of boiling treatment and OLA on MP gel WHC (**b**) and T_2_ relaxation time: (**c**) un-boiled and (**d**) boiled for 5 min. Different capital letters (a–d) indicate a significant difference (*p* < 0.05) between the control and boiling samples at different OLA concentrations.

The MP gel prepared by boiling and varying concentrations of OLA is shown in Figure 6a. Compared with the control group, the boiling treatment led to obvious water precipitation at the bottom of the gel, which was positively correlated with OLA concentration.

#### 3.4.1. Gel WHC

The WHC reflected the water content in the three-dimensional network formed by the MP gel and was related to the network structure of the protein gel. Figure 6b shows the changes in WHC of the MP gel under the boiling treatment and different OLA concentrations. Before the boiling treatment, as the OLA concentration increased to 5 mM, the gel WHC increased slightly and then declined with further increasing OLA concentration. This suggests that moderate oxidation could form a dense MP gel network, which increases the WHC of the protein gel by attracting more water into the gel network. However, excessive oxidation degrades the gel network structure and increases water mobility, which decreases the WHC [65].

After the boiling treatment, the WHC decreased with increasing OLA concentration. In addition, at the same OLA concentration, the WHC of all the boiling treatment gel was lower than that of the un-boiled gel, suggesting that the boiling treatment exacerbated the negative effects of OLA-induced MP oxidation on the network structure of the protein gel. The high-temperature boiling treatment disrupted the cross-linking between proteins, leading to the breakage of part of the gel network structure, causing the transfer of free water from the inside to the outside of the gel network [66], reducing the water binding force and, thus, exhibiting a worse WHC.

#### 3.4.2. Water Distribution

To further explore the distribution and movement of water in the MP gel system, the T_2_ spectrum of the gel was measured. T_21_, T_22_, and T_23_ represented the mobility of bound water, immobile water, and free water, respectively. T_21_ was the water that was closely bound to the protein through molecular forces, T_22_ was the most important water component in the gel, and T_23_ was the most easily lost water in the MP gel system [67]. PT_21_, PT_22_, and PT_23_ represented the proportion of water of different components in the total moisture of the gel.

Figure 6c shows the distribution of different forms of water in the MP gel system before the boiling treatment. Compared with non-oxidized MP, the T_22_ and T_23_ values of the MP gel increased with increasing OLA concentration. With increasing OLA concentration, PT_22_ increased when the OLA concentration was <5 mM and then decreased (OLA concentration > 5 mM), while PT_23_ showed the opposite trend (Figure S1a). The results show that, under moderate oxidation intensity, OLA strengthened the combination of water molecules in the gel network structure, reducing water fluidity. Further oxidation, however, caused a decrease in fixed water content and an increase in free water content. This was related to the transfer of water between macromolecules and the change in hydrogen bonding between protein and water [68].

As shown in Figure 6d, after boiling, the T_23_ relaxation time of the MP gel shortened with increasing OLA concentration, indicating that boiling further altered the water state in the MP gel system. Compared to the un-boiled MP gel, the P_22_ of the boiled gel decreased while P_23_ increased (Figure S1b). Both the shorter relaxation time and lower immobile water amount correlated with the reduced WHC [37]. After boiling, immobile water in the gel converted to free water, and the high fluidity of the free water decreased the WHC (Figure 6b).

## 4. Conclusions

The boiling treatment enhanced OLA’s capacity to oxidize MP in a dose-dependent manner, leading to significant changes in the physical and chemical properties as well as the structure of MP, which in turn affected the water retention of the MP gel. In detail, excessive oxidation caused by boiling reduced the WHC of the MP gel, decreased the amount of immobile water, and increased the amount of free water. The effect of protein oxidation on the water retention of fish is essential in determining its overall quality. This study provides a theoretical basis for improving the processing technology of water-boiled fish by decreasing protein oxidation, offering valuable insights into improving the quality and nutritional value of cooked fish products, and promoting healthy consumption of aquatic products.

## Figures and Tables

**Table 1:** Effect of boiling treatment and OLA concentration on the amino acid content of MP.

Amino Acids/	Concentration of OLA/(mM)									
(mg/g Protein)	0	2.5	5	7.5	10	0 + Boiling	2.5 + Boiling	5 + Boiling	7.5 + Boiling	10 + Boiling
Asp	16.989 ± 0.402 ^a^	14.802 ± 0.571 ^b^	14.617 ± 0.700 ^bc^	13.852 ± 0.174 ^bcd^	12.558 ± 0.530 ^e^	15.924 ± 0.030 ^a^	13.820 ± 0.128 ^bcd^	13.589 ± 0.429 ^cde^	13.065 ± 0.076 ^de^	9.749 ± 0.962 ^f^
Glu	31.733 ± 0.942 ^a^	28.436 ± 1.210 ^bc^	27.294 ± 1.785 ^cd^	26.752 ± 0.765 ^cde^	24.683 ± 1.124 ^de^	30.514 ± 0.926 ^ab^	27.056 ± 2.162 ^cde^	25.623 ± 1.078 ^cde^	24.102 ± 0.377 ^e^	21.031 ± 1.500 ^f^
Ser	7.383 ± 0.260 ^a^	6.643 ± 0.472 ^b^	6.207 ± 0.327 ^bc^	6.038 ± 0.091 ^bcd^	5.656 ± 0.224 ^cd^	6.756 ± 0.276 ^ab^	6.054 ± 0.402 ^bcd^	5.637 ± 0.056 ^cd^	5.406 ± 0.057 ^d^	4.558 ± 0.424 ^e^
His	3.009 ± 0.101 ^a^	2.685 ± 0.141 ^bc^	2.579 ± 0.144 ^bcd^	2.526 ± 0.030 ^bcde^	2.408 ± 0.122 ^cde^	2.792 ± 0.190 ^ab^	2.543 ± 0.107 ^bcd^	2.320 ± 0.046 ^de^	2.229 ± 0.031 ^e^	1.808 ± 0.202 ^f^
Gly	7.532 ± 0.286 ^a^	6.424 ± 0.218 ^b^	5.939 ± 0.412 ^bcd^	5.655 ± 0.138 ^cde^	5.176 ± 0.284 ^ef^	6.268 ± 0.281 ^bc^	5.400 ± 0.237 ^def^	5.092 ± 0.093 ^ef^	4.980 ± 0.074 ^f^	4.161 ± 0.404 ^g^
Thr	8.814 ± 1.217 ^a^	8.321 ± 0.089 ^ab^	7.871 ± 0.710 ^ab^	7.851 ± 0.090 ^ab^	6.983 ± 0.098 ^bc^	8.085 ± 0.402 ^ab^	8.149 ± 0.930 ^ab^	7.441 ± 0.305 ^abc^	7.262 ± 0.482 ^bc^	6.117 ± 0.507 ^c^
Arg	10.716 ± 0.248 ^a^	9.137 ± 0.043 ^bc^	8.927 ± 0.547 ^bcd^	8.696 ± 0.098 ^cde^	8.125 ± 0.378 ^de^	9.738 ± 0.224 ^b^	8.611 ± 0.477 ^cde^	8.123 ± 0.026 ^de^	7.870 ± 0.102 ^e^	6.497 ± 0.661 ^f^
Ala	9.830 ± 0.344 ^a^	8.496 ± 0.154 ^b^	8.292 ± 0.514 ^bc^	8.071 ± 0.152 ^bc^	7.451 ± 0.330 ^c^	8.782 ± 0.083 ^b^	7.938 ± 0.560 ^bc^	7.452 ± 0.005 ^c^	7.387 ± 0.485 ^c^	5.922 ± 0.589 ^d^
Tyr	6.386 ± 0.088 ^a^	5.554 ± 0.337 ^bc^	5.403 ± 0.257 ^cd^	5.210 ± 0.052 ^cd^	5.038 ± 0.284 ^cd^	6.040 ± 0.302 ^ab^	5.297 ± 0.455 ^cd^	4.929 ± 0.032 ^d^	4.836 ± 0.102 ^d^	4.064 ± 0.204 ^e^
Cys	1.959 ± 0.235 ^a^	1.766 ± 0.023 ^ab^	1.642 ± 0.123 ^abc^	1.619 ± 0.086 ^abc^	1.468 ± 0.033 ^bcd^	1.734 ± 0.170 ^ab^	1.682 ± 0.170 ^ab^	1.534 ± 0.119 ^bcd^	1.263 ± 0.216 ^cd^	1.218 ± 0.229 ^d^
Val	7.910 ± 0.187 ^a^	6.863 ± 0.214 ^b^	6.703 ± 0.354 ^bc^	6.488 ± 0.124 ^bc^	6.082 ± 0.291 ^c^	7.193 ± 0.234 ^b^	6.485 ± 0.375 ^bc^	6.097 ± 0.050 ^c^	6.036 ± 0.371 ^c^	4.857 ± 0.477 ^d^
Met	4.509 ± 0.416 ^a^	3.001 ± 0.006 ^bc^	2.560 ± 0.064 ^cd^	2.252 ± 0.098 ^d^	2.235 ± 0.087 ^d^	3.287 ± 0.419 ^b^	2.547 ± 0.423 ^cd^	2.207 ± 0.421 ^d^	1.821 ± 0.320 ^de^	1.450 ± 0.330 ^e^
Phe	5.963 ± 0.120 ^a^	5.035 ± 0.025 ^b^	4.823 ± 0.127 ^b^	4.885 ± 0.094 ^b^	4.705 ± 0.352 ^b^	5.701 ± 0.272 ^a^	4.952 ± 0.344 ^b^	4.655 ± 0.089 ^b^	4.499 ± 0.148 ^b^	3.762 ± 0.313 ^c^
Ile	7.761 ± 0.419 ^a^	6.997 ± 0.253 ^bc^	6.557 ± 0.118 ^cd^	6.632 ± 0.097 ^cd^	6.122 ± 0.213 ^de^	7.310 ± 0.013 ^ab^	6.599 ± 0.343 ^cd^	6.261 ± 0.115 ^de^	5.870 ± 0.116 ^e^	4.947 ± 0.555 ^f^
Leu	13.835 ± 0.796 ^a^	12.165 ± 0.697 ^bc^	11.832 ± 0.986 ^bcd^	11.407 ± 0.018 ^bcd^	10.610 ± 0.504 ^d^	12.704 ± 0.496 ^ab^	11.410 ± 0.754 ^bcd^	10.766 ± 0.192 ^cd^	10.391 ± 0.406 ^d^	8.629 ± 0.767 ^e^
Iys	14.406 ± 0.023 ^a^	12.511 ± 0.588 ^bc^	11.862 ± 0.605 ^cd^	11.496 ± 0.441 ^cd^	11.081 ± 0.490 ^d^	13.220 ± 0.681 ^b^	11.726 ± 0.653 ^cd^	11.201 ± 0.040 ^d^	10.859 ± 0.123 ^d^	8.597 ± 0.815 ^e^
Pro	5.338 ± 0.664 ^a^	4.463 ± 0.033 ^ab^	4.171 ± 0.257 ^b^	4.044 ± 0.131 ^b^	3.737 ± 0.109 ^bc^	4.600 ± 0.680 ^ab^	3.988 ± 0.542 ^b^	3.715 ± 0.122 ^bc^	3.671 ± 0.141 ^bc^	2.868 ± 0.445 ^c^

Values represent the average ± SD; SD stands for standard deviation of mean. The superscripts (a–g) of different lowercase letters in the same row indicate significant differences (*p* < 0.05).

## Data Availability

The original contributions presented in the study are included in the article/Supplementary Materials, further inquiries can be directed to the corresponding author.

## Supplementary Materials

The following supporting information can be downloaded at: https://www.mdpi.com/article/10.3390/foods13244153/s1, Figure S1. Effect of boiling treatment and OLA concentration on the proportion of peak areas of MP gel system, (a) was un-boiled, (b) was boiled for 5 min.
