# Supplementary material for: Effect of Boiling Treatment on Linoleic Acid-Induced Oxidation of Myofibrillar Protein in Grass Carp
Source: Foods. 2024 Dec 22;13(24):4153. doi: 10.3390/foods13244153 (PMC11675559; doi:10.3390/foods13244153)
Supplement: Supplementary file 1 [file foods-13-04153-s001.zip › foods-3305173-supplementary.pdf]

Supplementary Material

3.4.2 Water distribution

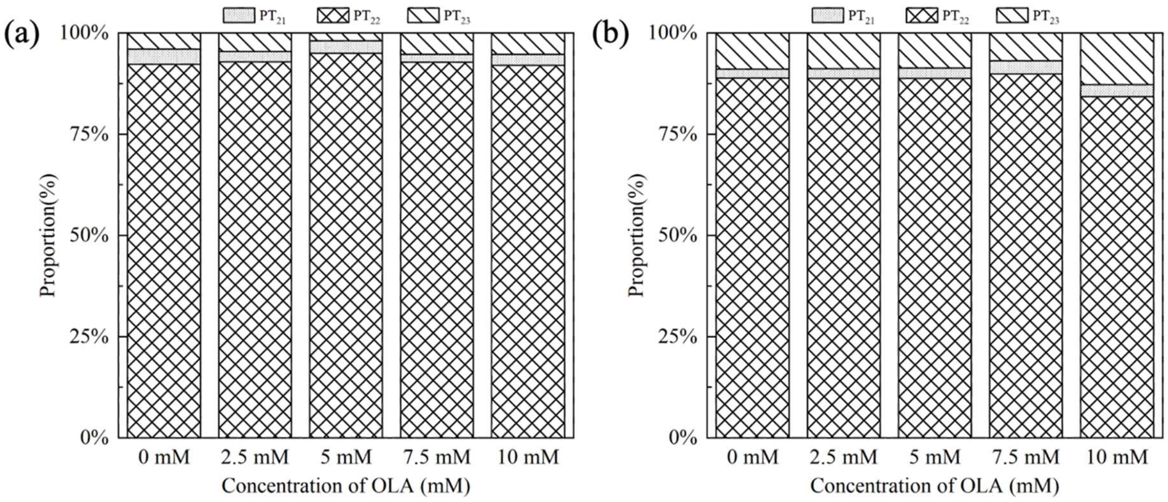

Figure. S1. Effect of boiling treatment and OLA concentration on the proportion of peak areas of MP gel system,

(a) was un-boiled, (b) was boiled for 5 min.
